# Supplementary material for: Correlation of the antibacterial activity of commercial manuka and Leptospermum honeys from Australia and New Zealand with methylglyoxal content and other physicochemical characteristics
Source: PLoS One. 2022 Jul 28;17(7):e0272376. doi: 10.1371/journal.pone.0272376 (PMC9333225; doi:10.1371/journal.pone.0272376)
Supplement: S1 Table — (DOCX) [file pone.0272376.s001.docx]

Supplementary Table 1. Concentration of honey at which the OD was reduced by 50% and 90%, relative to the positive growth control

|  | *S.aureus*  ATCC 29213 | |  | *E. faecalis*  ATCC 29212 | |  | *E. coli*  ATCC 25922 | |  | *P. aeruginosa*  ATCC 27853 | |
| --- | --- | --- | --- | --- | --- | --- | --- | --- | --- | --- | --- |
|  | 50% | 90% |  | 50% | 90% |  | 50% | 90% |  | 50% | 90% |
| MN01 | 7 | 12 |  | 23 | 25 |  | 10 | 16 |  | 18 | 19 |
| MN02 | 25 | >30 |  | 28 | >30 |  | 14 | 28 |  | 19 | 22 |
| MN03 | 4 | 4 |  | 10 | 10 |  | 6 | 6 |  | 13 | 14 |
| MN04 | 8 | 8 |  | 24 | 28 |  | 11 | 14 |  | 14 | 14 |
| MN05 | 9 | 12 |  | 20 | 22 |  | 7 | 17 |  | 20 | 24 |
| MN06 | 14 | 16 |  | 25 | 27 |  | 11 | 24 |  | 22 | 24 |
| MN07 | 3 | 8 |  | 16 | 18 |  | 10 | 10 |  | 17 | 20 |
| MN08 | 6 | 8 |  | 14 | 16 |  | 6 | 10 |  | 15 | 16 |
| MN09 | 7 | 8 |  | 25 | >30 |  | 11 | 25 |  | 12 | 14 |
| MN10 | 3 | 4 |  | 12 | 12 |  | 6 | 6 |  | 13 | 16 |
| MN11 | 8 | 8 |  | 16 | 18 |  | 8 | 10 |  | 16 | 18 |
| MN12 | 6 | 6 |  | 14 | 16 |  | 7 | 8 |  | 15 | 16 |
| MN13 | 10 | 12 |  | 21 | 24 |  | 10 | 14 |  | 18 | 23 |
| MN14 | 16 | 23 |  | 27 | >30 |  | 9 | 28 |  | 20 | 24 |
| MN15 | 17 | 24 |  | 29 | >30 |  | 14 | 26 |  | 19 | 23 |
| MN16 | 5 | 6 |  | 14 | 16 |  | 8 | 8 |  | 16 | 19 |
| MN17 | 14 | 24 |  | 25 | 30 |  | 12 | 27 |  | 16 | 18 |
| MN18 | 9 | 10 |  | 27 | 30 |  | 13 | 18 |  | 15 | 17 |
| MN19 | 4 | 6 |  | 27 | 30 |  | 14 | 23 |  | 11 | 13 |
| MN20 | 10 | 12 |  | 25 | 30 |  | 12 | 19 |  | 15 | 16 |
| MN21 | 7 | 8 |  | 16 | 17 |  | 9 | 12 |  | 19 | 24 |
| MN22 | 5 | 5 |  | 10 | 12 |  | 7 | 8 |  | 15 | 18 |
| MN23 | 4 | 6 |  | 13 | 14 |  | 7 | 10 |  | 15 | 16 |
| MN24 | 17 | 20 |  | 23 | 30 |  | 11 | 24 |  | 20 | 24 |
| MN25 | 8 | 8 |  | 18 | 20 |  | 10 | 12 |  | 14 | 16 |
| MN26 | 14 | 14 |  | 26 | 30 |  | 11 | 18 |  | 18 | 22 |
| MN27 | 6 | 8 |  | 15 | 16 |  | 10 | 11 |  | 18 | 20 |
| MN28 | 4 | 4 |  | 23 | 29 |  | 14 | 20 |  | 10 | 12 |
| MN29 | 4 | 6 |  | 17 | 22 |  | 4 | 22 |  | 10 | 10 |
| ART | 21 | >30 |  | 26 | >30 |  | 16 | 30 |  | 19 | 24 |
